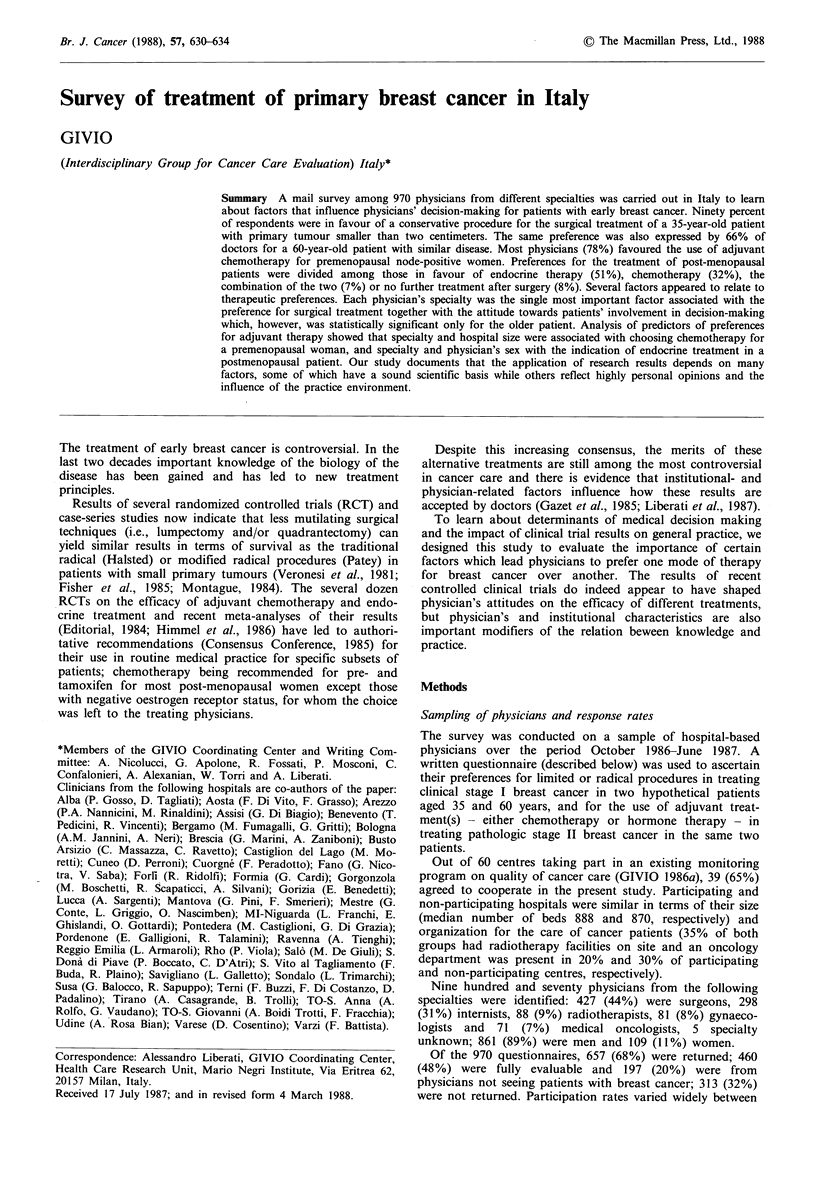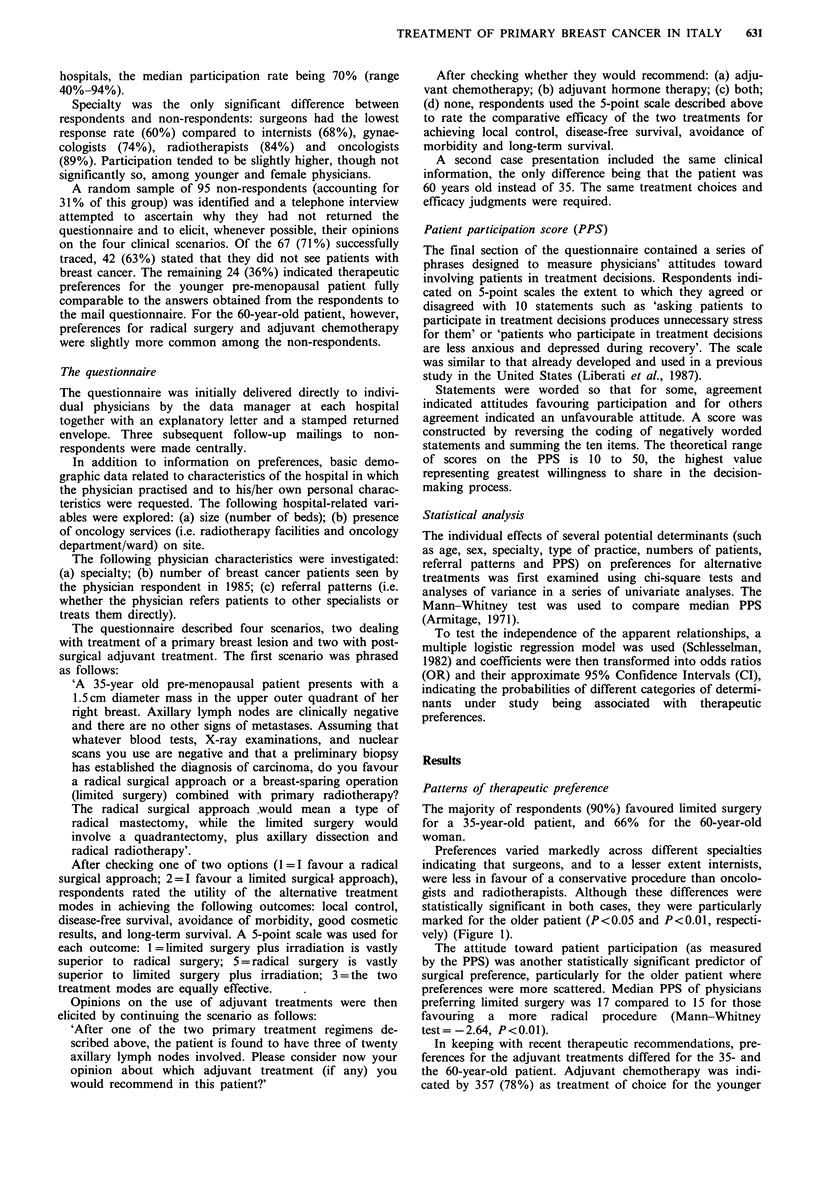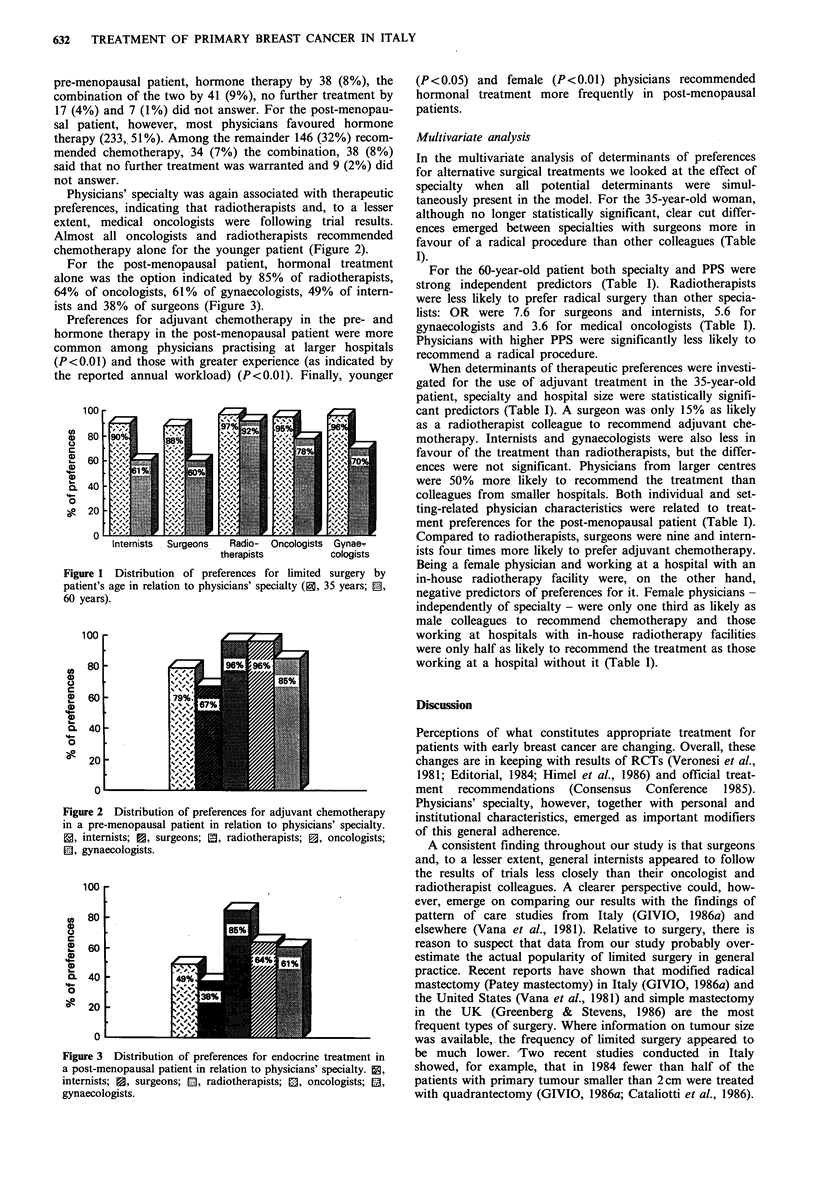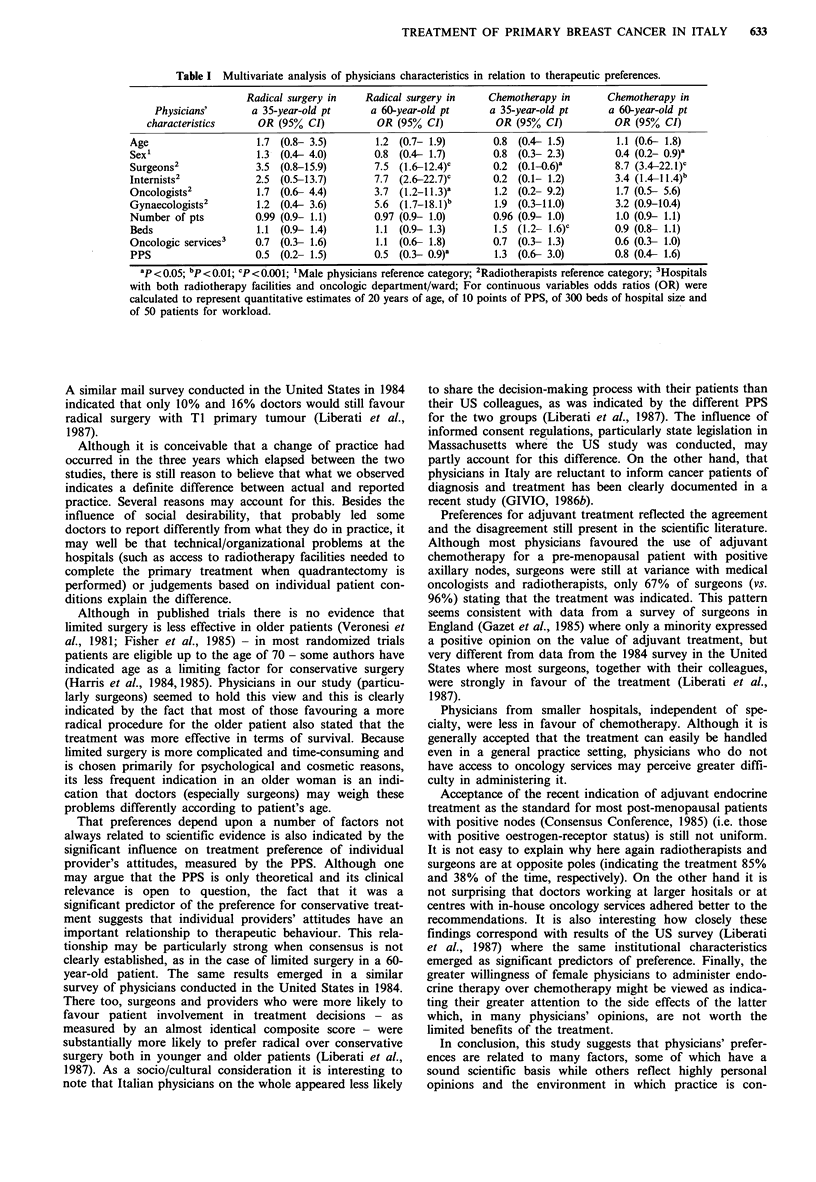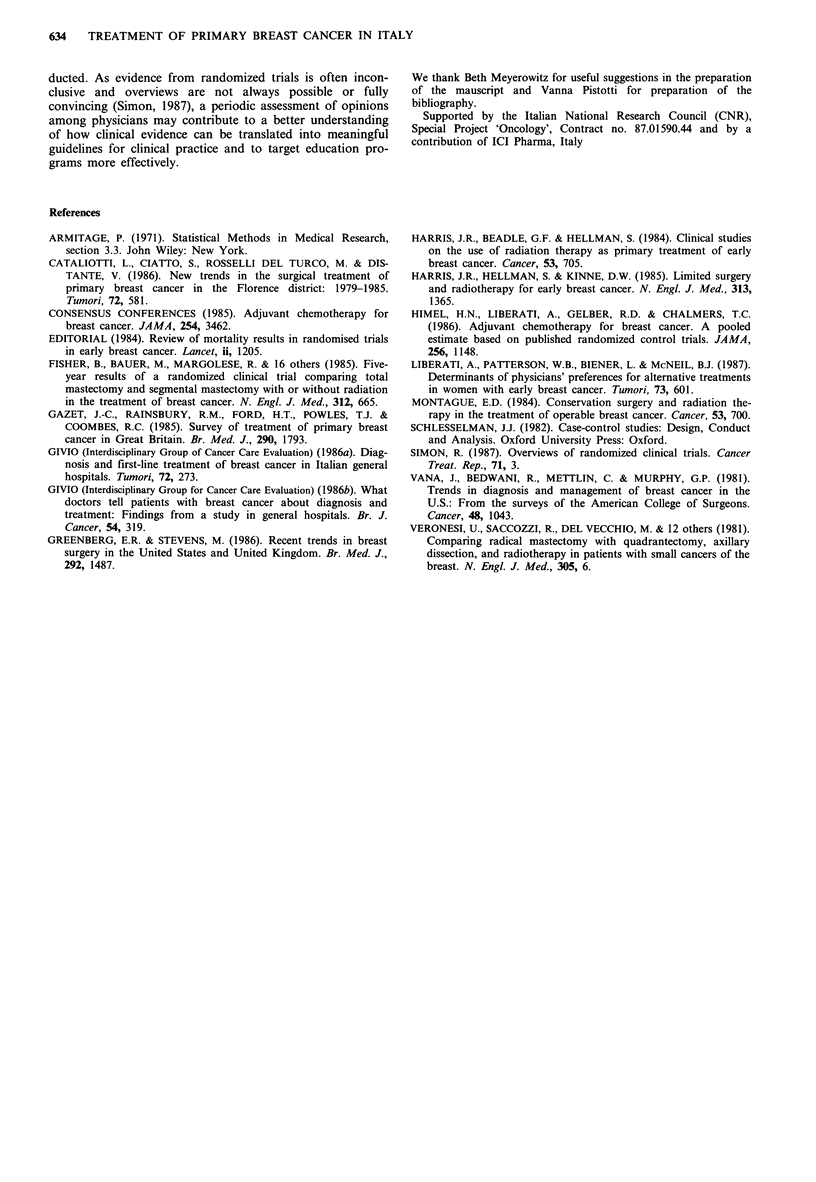# Survey of treatment of primary breast cancer in Italy. GIVIO (Interdisciplinary Group for Cancer Care Evaluation).

**DOI:** 10.1038/bjc.1988.144

**Published:** 1988-06

**Authors:** 

## Abstract

A mail survey among 970 physicians from different specialties was carried out in Italy to learn about factors that influence physicians' decision-making for patients with early breast cancer. Ninety percent of respondents were in favour of a conservative procedure for the surgical treatment of a 35-year-old patient with primary tumour smaller than two centimeters. The same preference was also expressed by 66% of doctors for a 60-year-old patient with similar disease. Most physicians (78%) favoured the use of adjuvant chemotherapy for premenopausal node-positive women. Preferences for the treatment of post-menopausal patients were divided among those in favour of endocrine therapy (51%), chemotherapy (32%), the combination of the two (7%) or no further treatment after surgery (8%). Several factors appeared to relate to therapeutic preferences. Each physician's specialty was the single most important factor associated with the preference for surgical treatment together with the attitude towards patients' involvement in decision-making which, however, was statistically significant only for the older patient. Analysis of predictors of preferences for adjuvant therapy showed that specialty and hospital size were associated with choosing chemotherapy for a premenopausal woman, and specialty and physician's sex with the indication of endocrine treatment in a postmenopausal patient. Our study documents that the application of research results depends on many factors, some of which have a sound scientific basis while others reflect highly personal opinions and the influence of the practice environment.


*Br. J. Cancer* (1988), **57**, 630–634 © The Macmillan Press, Ltd., 1988

### Survey of treatment of primary breast cancer in Italy

GIVIO

*(Interdisciplinary Group for Cancer Care Evaluation) Italy**

**Summary** A mail survey among 970 physicians from different specialties was carried out in Italy to learn about factors that influence physicians' decision-making for patients with early breast cancer. Ninety percent of respondents were in favour of a conservative procedure for the surgical treatment of a 35-year-old patient with primary tumour smaller than two centimeters. The same preference was also expressed by 66% of doctors for a 60-year-old patient with similar disease. Most physicians (78%) favoured the use of adjuvant chemotherapy for premenopausal node-positive women. Preferences for the treatment of post-menopausal patients were divided among those in favour of endocrine therapy (51%), chemotherapy (32%), the combination of the two (7%) or no further treatment after surgery (8%). Several factors appeared to relate to therapeutic preferences. Each physician's specialty was the single most important factor associated with the preference for surgical treatment together with the attitude towards patients' involvement in decision-making which, however, was statistically significant only for the older patient. Analysis of predictors of preferences for adjuvant therapy showed that specialty and hospital size were associated with choosing chemotherapy for a premenopausal woman, and specialty and physician's sex with the indication of endocrine treatment in a postmenopausal patient. Our study documents that the application of research results depends on many factors, some of which have a sound scientific basis while others reflect highly personal opinions and the influence of the practice environment.

The treatment of early breast cancer is controversial. In the last two decades important knowledge of the biology of the disease has been gained and has led to new treatment principles.

Results of several randomized controlled trials (RCT) and case-series studies now indicate that less mutilating surgical techniques (i.e., lumpectomy and/or quadrantectomy) can yield similar results in terms of survival as the traditional radical (Halsted) or modified radical procedures (Patey) in patients with small primary tumours (Veronesi *et al.*, 1981; Fisher *et al.*, 1985; Montague, 1984). The several dozen RCTs on the efficacy of adjuvant chemotherapy and endocrine treatment and recent meta-analyses of their results (Editorial, 1984; Himmel *et al.*, 1986) have led to authoritative recommendations (Consensus Conference, 1985) for their use in routine medical practice for specific subsets of patients; chemotherapy being recommended for pre- and tamoxifen for most post-menopausal women except those with negative oestrogen receptor status, for whom the choice was left to the treating physicians.

*Members of the GIVIO Coordinating Center and Writing Committee: A. Nicolucci, G. Apolone, R. Fossati, P. Mosconi, C. Confalonieri, A. Alexanian, W. Torri and A. Liberati.
Clinicians from the following hospitals are co-authors of the paper: Alba (P. Gosso, D. Tagliati); Aosta (F. Di Vito, F. Grasso); Arezzo (P.A. Nannicini, M. Rinaldini); Assisi (G. Di Biagio); Benevento (T. Pedicini, R. Vincenti); Bergamo (M. Fumagalli, G. Gritti); Bologna (A.M. Jannini, A. Neri); Brescia (G. Marini, A. Zaniboni); Busto Arsizio (C. Massazza, C. Ravetto); Castiglion del Lago (M. Moretti); Cuneo (D. Perroni); Cuorgné (F. Peradotto); Fano (G. Nicotra, V. Saba); Forlì (R. Ridolfi); Formia (G. Cardi); Gorgonzola (M. Boschetti, R. Scapaticci, A. Silvani); Gorizia (E. Benedetti); Lucca (A. Sargenti); Mantova (G. Pini, F. Smerieri); Mestre (G. Conte, L. Griggio, O. Nascimben); MI-Niguarda (L. Franchi, E. Ghislandi, O. Gottardi); Pontedera (M. Castiglioni, G. Di Grazia); Pordenone (E. Galligioni, R. Talamini); Ravenna (A. Tienghi); Reggio Emilia (L. Armaroli); Rho (P. Viola); Salò (M. De Giuli); S. Donà di Piave (P. Boccato, C. D'Atri); S. Vito al Tagliamento (F. Buda, R. Plaino); Savigliano (L. Galletto); Sondalo (L. Trimarchi); Susa (G. Balocco, R. Sapuppo); Terni (F. Buzzi, F. Di Costanzo, D. Padalino); Tirano (A. Casagrande, B. Trolli); TO-S. Anna (A. Rolfo, G. Vaudano); TO-S. Giovanni (A. Boidi Trotti, F. Fracchia); Udine (A. Rosa Bian); Varese (D. Cosentino); Varzi (F. Battista).

Correspondence: Alessandro Liberati, GIVIO Coordinating Center, Health Care Research Unit, Mario Negri Institute, Via Eritrea 62, 20157 Milan, Italy.
Received 17 July 1987; and in revised form 4 March 1988.

Despite this increasing consensus, the merits of these alternative treatments are still among the most controversial in cancer care and there is evidence that institutional- and physician-related factors influence how these results are accepted by doctors (Gazet *et al.*, 1985; Liberati *et al.*, 1987).

To learn about determinants of medical decision making and the impact of clinical trial results on general practice, we designed this study to evaluate the importance of certain factors which lead physicians to prefer one mode of therapy for breast cancer over another. The results of recent controlled clinical trials do indeed appear to have shaped physician's attitudes on the efficacy of different treatments, but physician's and institutional characteristics are also important modifiers of the relation beween knowledge and practice.

### Methods

### *Sampling of physicians and response rates*

The survey was conducted on a sample of hospital-based physicians over the period October 1986–June 1987. A written questionnaire (described below) was used to ascertain their preferences for limited or radical procedures in treating clinical stage I breast cancer in two hypothetical patients aged 35 and 60 years, and for the use of adjuvant treatment(s) – either chemotherapy or hormone therapy – in treating pathologic stage II breast cancer in the same two patients.

Out of 60 centres taking part in an existing monitoring program on quality of cancer care (GIVIO 1986*a*), 39 (65%) agreed to cooperate in the present study. Participating and non-participating hospitals were similar in terms of their size (median number of beds 888 and 870, respectively) and organization for the care of cancer patients (35% of both groups had radiotherapy facilities on site and an oncology department was present in 20% and 30% of participating and non-participating centres, respectively).

Nine hundred and seventy physicians from the following specialties were identified: 427 (44%) were surgeons, 298 (31%) internists, 88 (9%) radiotherapists, 81 (8%) gynaecologists and 71 (7%) medical oncologists, 5 specialty unknown; 861 (89%) were men and 109 (11%) women.

Of the 970 questionnaires, 657 (68%) were returned; 460 (48%) were fully evaluable and 197 (20%) were from physicians not seeing patients with breast cancer; 313 (32%) were not returned. Participation rates varied widely between hospitals, the median participation rate being 70% (range 40%–94%).

Specialty was the only significant difference between respondents and non-respondents: surgeons had the lowest response rate (60%) compared to internists (68%), gynaecologists (74%), radiotherapists (84%) and oncologists (89%). Participation tended to be slightly higher, though not significantly so, among younger and female physicians.

A random sample of 95 non-respondents (accounting for 31% of this group) was identified and a telephone interview attempted to ascertain why they had not returned the questionnaire and to elicit, whenever possible, their opinions on the four clinical scenarios. Of the 67 (71%) successfully traced, 42 (63%) stated that they did not see patients with breast cancer. The remaining 24 (36%) indicated therapeutic preferences for the younger pre-menopausal patient fully comparable to the answers obtained from the respondents to the mail questionnaire. For the 60-year-old patient, however, preferences for radical surgery and adjuvant chemotherapy were slightly more common among the non-respondents.

### *The questionnaire*

The questionnaire was initially delivered directly to individual physicians by the data manager at each hospital together with an explanatory letter and a stamped returned envelope. Three subsequent follow-up mailings to non-respondents were made centrally.

In addition to information on preferences, basic demographic data related to characteristics of the hospital in which the physician practised and to his/her own personal characteristics were requested. The following hospital-related variables were explored: (a) size (number of beds); (b) presence of oncology services (i.e. radiotherapy facilities and oncology department/ward) on site.

The following physician characteristics were investigated: (a) specialty; (b) number of breast cancer patients seen by the physician respondent in 1985; (c) referral patterns (i.e. whether the physician refers patients to other specialists or treats them directly).

The questionnaire described four scenarios, two dealing with treatment of a primary breast lesion and two with post-surgical adjuvant treatment. The first scenario was phrased as follows:

'A 35-year old pre-menopausal patient presents with a 1.5 cm diameter mass in the upper outer quadrant of her right breast. Axillary lymph nodes are clinically negative and there are no other signs of metastases. Assuming that whatever blood tests, X-ray examinations, and nuclear scans you use are negative and that a preliminary biopsy has established the diagnosis of carcinoma, do you favour a radical surgical approach or a breast-sparing operation (limited surgery) combined with primary radiotherapy? The radical surgical approach would mean a type of radical mastectomy, while the limited surgery would involve a quadrantectomy, plus axillary dissection and radical radiotherapy'.

After checking one of two options (1 = I favour a radical surgical approach; 2 = I favour a limited surgical approach), respondents rated the utility of the alternative treatment modes in achieving the following outcomes: local control, disease-free survival, avoidance of morbidity, good cosmetic results, and long-term survival. A 5-point scale was used for each outcome: 1 = limited surgery plus irradiation is vastly superior to radical surgery; 5 = radical surgery is vastly superior to limited surgery plus irradiation; 3 = the two treatment modes are equally effective.

Opinions on the use of adjuvant treatments were then elicited by continuing the scenario as follows:

'After one of the two primary treatment regimens described above, the patient is found to have three of twenty axillary lymph nodes involved. Please consider now your opinion about which adjuvant treatment (if any) you would recommend in this patient?'

After checking whether they would recommend: (a) adjuvant chemotherapy; (b) adjuvant hormone therapy; (c) both; (d) none, respondents used the 5-point scale described above to rate the comparative efficacy of the two treatments for achieving local control, disease-free survival, avoidance of morbidity and long-term survival.

A second case presentation included the same clinical information, the only difference being that the patient was 60 years old instead of 35. The same treatment choices and efficacy judgments were required.

### *Patient participation score (PPS)*

The final section of the questionnaire contained a series of phrases designed to measure physicians' attitudes toward involving patients in treatment decisions. Respondents indicated on 5-point scales the extent to which they agreed or disagreed with 10 statements such as 'asking patients to participate in treatment decisions produces unnecessary stress for them' or 'patients who participate in treatment decisions are less anxious and depressed during recovery'. The scale was similar to that already developed and used in a previous study in the United States (Liberati *et al.*, 1987).

Statements were worded so that for some, agreement indicated attitudes favouring participation and for others agreement indicated an unfavourable attitude. A score was constructed by reversing the coding of negatively worded statements and summing the ten items. The theoretical range of scores on the PPS is 10 to 50, the highest value representing greatest willingness to share in the decision-making process.

### *Statistical analysis*

The individual effects of several potential determinants (such as age, sex, specialty, type of practice, numbers of patients, referral patterns and PPS) on preferences for alternative treatments was first examined using chi-square tests and analyses of variance in a series of univariate analyses. The Mann–Whitney test was used to compare median PPS (Armitage, 1971).

To test the independence of the apparent relationships, a multiple logistic regression model was used (Schlesselman, 1982) and coefficients were then transformed into odds ratios (OR) and their approximate 95% Confidence Intervals (CI), indicating the probabilities of different categories of determinants under study being associated with therapeutic preferences.

### Results

### *Patterns of therapeutic preference*

The majority of respondents (90%) favoured limited surgery for a 35-year-old patient, and 66% for the 60-year-old woman.

Preferences varied markedly across different specialties indicating that surgeons, and to a lesser extent internists, were less in favour of a conservative procedure than oncologists and radiotherapists. Although these differences were statistically significant in both cases, they were particularly marked for the older patient ($P<0.05$ and $P<0.01$, respectively) (Figure 1).

The attitude toward patient participation (as measured by the PPS) was another statistically significant predictor of surgical preference, particularly for the older patient where preferences were more scattered. Median PPS of physicians preferring limited surgery was 17 compared to 15 for those favouring a more radical procedure (Mann–Whitney test = −2.64, $P<0.01$).

In keeping with recent therapeutic recommendations, preferences for the adjuvant treatments differed for the 35- and the 60-year-old patient. Adjuvant chemotherapy was indicated by 357 (78%) as treatment of choice for the younger pre-menopausal patient, hormone therapy by 38 (8%), the combination of the two by 41 (9%), no further treatment by 17 (4%) and 7 (1%) did not answer. For the post-menopausal patient, however, most physicians favoured hormone therapy (233, 51%). Among the remainder 146 (32%) recommended chemotherapy, 34 (7%) the combination, 38 (8%) said that no further treatment was warranted and 9 (2%) did not answer.

Physicians' specialty was again associated with therapeutic preferences, indicating that radiotherapists and, to a lesser extent, medical oncologists were following trial results. Almost all oncologists and radiotherapists recommended chemotherapy alone for the younger patient (Figure 2).

For the post-menopausal patient, hormonal treatment alone was the option indicated by 85% of radiotherapists, 64% of oncologists, 61% of gynaecologists, 49% of internists and 38% of surgeons (Figure 3).

Preferences for adjuvant chemotherapy in the pre- and hormone therapy in the post-menopausal patient were more common among physicians practising at larger hospitals ($P<0.01$) and those with greater experience (as indicated by the reported annual workload) ($P<0.01$). Finally, younger

**Figure 1** Distribution of preferences for limited surgery by patient's age in relation to physicians' specialty (▨, 35 years; ▤, 60 years).

**Figure 2** Distribution of preferences for adjuvant chemotherapy in a pre-menopausal patient in relation to physicians' specialty. ▨, internists; ▨, surgeons; ▤, radiotherapists; ▨, oncologists; ▤, gynaecologists.

**Figure 3** Distribution of preferences for endocrine treatment in a post-menopausal patient in relation to physicians' specialty. ▨, internists; ▨, surgeons; ▤, radiotherapists; ▨, oncologists; ▤, gynaecologists.

($P<0.05$) and female ($P<0.01$) physicians recommended hormonal treatment more frequently in post-menopausal patients.

*Multivariate analysis*

In the multivariate analysis of determinants of preferences for alternative surgical treatments we looked at the effect of specialty when all potential determinants were simultaneously present in the model. For the 35-year-old woman, although no longer statistically significant, clear cut differences emerged between specialties with surgeons more in favour of a radical procedure than other colleagues (Table I).

For the 60-year-old patient both specialty and PPS were strong independent predictors (Table I). Radiotherapists were less likely to prefer radical surgery than other specialists: OR were 7.6 for surgeons and internists, 5.6 for gynaecologists and 3.6 for medical oncologists (Table I). Physicians with higher PPS were significantly less likely to recommend a radical procedure.

When determinants of therapeutic preferences were investigated for the use of adjuvant treatment in the 35-year-old patient, specialty and hospital size were statistically significant predictors (Table I). A surgeon was only 15% as likely as a radiotherapist colleague to recommend adjuvant chemotherapy. Internists and gynaecologists were also less in favour of the treatment than radiotherapists, but the differences were not significant. Physicians from larger centres were 50% more likely to recommend the treatment than colleagues from smaller hospitals. Both individual and setting-related physician characteristics were related to treatment preferences for the post-menopausal patient (Table I). Compared to radiotherapists, surgeons were nine and internists four times more likely to prefer adjuvant chemotherapy. Being a female physician and working at a hospital with an in-house radiotherapy facility were, on the other hand, negative predictors of preferences for it. Female physicians – independently of specialty – were only one third as likely as male colleagues to recommend chemotherapy and those working at hospitals with in-house radiotherapy facilities were only half as likely to recommend the treatment as those working at a hospital without it (Table I).

### Discussion

Perceptions of what constitutes appropriate treatment for patients with early breast cancer are changing. Overall, these changes are in keeping with results of RCTs (Veronesi *et al.*, 1981; Editorial, 1984; Himel *et al.*, 1986) and official treatment recommendations (Consensus Conference 1985). Physicians' specialty, however, together with personal and institutional characteristics, emerged as important modifiers of this general adherence.

A consistent finding throughout our study is that surgeons and, to a lesser extent, general internists appeared to follow the results of trials less closely than their oncologist and radiotherapist colleagues. A clearer perspective could, however, emerge on comparing our results with the findings of pattern of care studies from Italy (GIVIO, 1986*a*) and elsewhere (Vana *et al.*, 1981). Relative to surgery, there is reason to suspect that data from our study probably overestimate the actual popularity of limited surgery in general practice. Recent reports have shown that modified radical mastectomy (Patey mastectomy) in Italy (GIVIO, 1986*a*) and the United States (Vana *et al.*, 1981) and simple mastectomy in the UK (Greenberg & Stevens, 1986) are the most frequent types of surgery. Where information on tumour size was available, the frequency of limited surgery appeared to be much lower. Two recent studies conducted in Italy showed, for example, that in 1984 fewer than half of the patients with primary tumour smaller than 2 cm were treated with quadrantectomy (GIVIO, 1986*a*; Cataliotti *et al.*, 1986).

**Table I** Multivariate analysis of physicians characteristics in relation to therapeutic preferences.

| *Physicians' characteristics* | *Radical surgery in a 35-year-old pt OR (95% CI)* | *Radical surgery in a 60-year-old pt OR (95% CI)* | *Chemotherapy in a 35-year-old pt OR (95% CI)* | *Chemotherapy in a 60-year-old pt OR (95% CI)* |
|---|---|---|---|---|
| Age | 1.7 (0.8– 3.5) | 1.2 (0.7– 1.9) | 0.8 (0.4– 1.5) | 1.1 (0.6– 1.8) |
| Sex[1] | 1.3 (0.4– 4.0) | 0.8 (0.4– 1.7) | 0.8 (0.3– 2.3) | 0.4 (0.2– 0.9)[a] |
| Surgeons[2] | 3.5 (0.8–15.9) | 7.5 (1.6–12.4)[c] | 0.2 (0.1–0.6)[a] | 8.7 (3.4–22.1)[c] |
| Internists[2] | 2.5 (0.5–13.7) | 7.7 (2.6–22.7)[c] | 0.2 (0.1– 1.2) | 3.4 (1.4–11.4)[b] |
| Oncologists[2] | 1.7 (0.6– 4.4) | 3.7 (1.2–11.3)[a] | 1.2 (0.2– 9.2) | 1.7 (0.5– 5.6) |
| Gynaecologists[2] | 1.2 (0.4– 3.6) | 5.6 (1.7–18.1)[b] | 1.9 (0.3–11.0) | 3.2 (0.9–10.4) |
| Number of pts | 0.99 (0.9– 1.1) | 0.97 (0.9– 1.0) | 0.96 (0.9– 1.0) | 1.0 (0.9– 1.1) |
| Beds | 1.1 (0.9– 1.4) | 1.1 (0.9– 1.3) | 1.5 (1.2– 1.6)[c] | 0.9 (0.8– 1.1) |
| Oncologic services[3] | 0.7 (0.3– 1.6) | 1.1 (0.6– 1.8) | 0.7 (0.3– 1.3) | 0.6 (0.3– 1.0) |
| PPS | 0.5 (0.2– 1.5) | 0.5 (0.3– 0.9)[a] | 1.3 (0.6– 3.0) | 0.8 (0.4– 1.6) |

[a]$P<0.05$; [b]$P<0.01$; [c]$P<0.001$; [1]Male physicians reference category; [2]Radiotherapists reference category; [3]Hospitals with both radiotherapy facilities and oncologic department/ward; For continuous variables odds ratios (OR) were calculated to represent quantitative estimates of 20 years of age, of 10 points of PPS, of 300 beds of hospital size and of 50 patients for workload.

A similar mail survey conducted in the United States in 1984 indicated that only 10% and 16% doctors would still favour radical surgery with T1 primary tumour (Liberati *et al.*, 1987).

Although it is conceivable that a change of practice had occurred in the three years which elapsed between the two studies, there is still reason to believe that what we observed indicates a definite difference between actual and reported practice. Several reasons may account for this. Besides the influence of social desirability, that probably led some doctors to report differently from what they do in practice, it may well be that technical/organizational problems at the hospitals (such as access to radiotherapy facilities needed to complete the primary treatment when quadrantectomy is performed) or judgements based on individual patient conditions explain the difference.

Although in published trials there is no evidence that limited surgery is less effective in older patients (Veronesi *et al.*, 1981; Fisher *et al.*, 1985) – in most randomized trials patients are eligible up to the age of 70 – some authors have indicated age as a limiting factor for conservative surgery (Harris *et al.*, 1984, 1985). Physicians in our study (particularly surgeons) seemed to hold this view and this is clearly indicated by the fact that most of those favouring a more radical procedure for the older patient also stated that the treatment was more effective in terms of survival. Because limited surgery is more complicated and time-consuming and is chosen primarily for psychological and cosmetic reasons, its less frequent indication in an older woman is an indication that doctors (especially surgeons) may weigh these problems differently according to patient's age.

That preferences depend upon a number of factors not always related to scientific evidence is also indicated by the significant influence on treatment preference of individual provider's attitudes, measured by the PPS. Although one may argue that the PPS is only theoretical and its clinical relevance is open to question, the fact that it was a significant predictor of the preference for conservative treatment suggests that individual providers' attitudes have an important relationship to therapeutic behaviour. This relationship may be particularly strong when consensus is not clearly established, as in the case of limited surgery in a 60-year-old patient. The same results emerged in a similar survey of physicians conducted in the United States in 1984. There too, surgeons and providers who were more likely to favour patient involvement in treatment decisions – as measured by an almost identical composite score – were substantially more likely to prefer radical over conservative surgery both in younger and older patients (Liberati *et al.*, 1987). As a socio/cultural consideration it is interesting to note that Italian physicians on the whole appeared less likely to share the decision-making process with their patients than their US colleagues, as was indicated by the different PPS for the two groups (Liberati *et al.*, 1987). The influence of informed consent regulations, particularly state legislation in Massachusetts where the US study was conducted, may partly account for this difference. On the other hand, that physicians in Italy are reluctant to inform cancer patients of diagnosis and treatment has been clearly documented in a recent study (GIVIO, 1986*b*).

Preferences for adjuvant treatment reflected the agreement and the disagreement still present in the scientific literature. Although most physicians favoured the use of adjuvant chemotherapy for a pre-menopausal patient with positive axillary nodes, surgeons were still at variance with medical oncologists and radiotherapists, only 67% of surgeons (*vs.* 96%) stating that the treatment was indicated. This pattern seems consistent with data from a survey of surgeons in England (Gazet *et al.*, 1985) where only a minority expressed a positive opinion on the value of adjuvant treatment, but very different from data from the 1984 survey in the United States where most surgeons, together with their colleagues, were strongly in favour of the treatment (Liberati *et al.*, 1987).

Physicians from smaller hospitals, independent of specialty, were less in favour of chemotherapy. Although it is generally accepted that the treatment can easily be handled even in a general practice setting, physicians who do not have access to oncology services may perceive greater difficulty in administering it.

Acceptance of the recent indication of adjuvant endocrine treatment as the standard for most post-menopausal patients with positive nodes (Consensus Conference, 1985) (i.e. those with positive oestrogen-receptor status) is still not uniform. It is not easy to explain why here again radiotherapists and surgeons are at opposite poles (indicating the treatment 85% and 38% of the time, respectively). On the other hand it is not surprising that doctors working at larger hositals or at centres with in-house oncology services adhered better to the recommendations. It is also interesting how closely these findings correspond with results of the US survey (Liberati *et al.*, 1987) where the same institutional characteristics emerged as significant predictors of preference. Finally, the greater willingness of female physicians to administer endocrine therapy over chemotherapy might be viewed as indicating their greater attention to the side effects of the latter which, in many physicians' opinions, are not worth the limited benefits of the treatment.

In conclusion, this study suggests that physicians' preferences are related to many factors, some of which have a sound scientific basis while others reflect highly personal opinions and the environment in which practice is conducted. As evidence from randomized trials is often inconclusive and overviews are not always possible or fully convincing (Simon, 1987), a periodic assessment of opinions among physicians may contribute to a better understanding of how clinical evidence can be translated into meaningful guidelines for clinical practice and to target education programs more effectively.

We thank Beth Meyerowitz for useful suggestions in the preparation of the mauscript and Vanna Pistotti for preparation of the bibliography.

Supported by the Italian National Research Council (CNR), Special Project 'Oncology', Contract no. 87.01590.44 and by a contribution of ICI Pharma, Italy

### References

ARMITAGE, P. (1971). Statistical Methods in Medical Research, section 3.3. John Wiley: New York.

CATALIOTTI, L., CIATTO, S., ROSSELLI DEL TURCO, M. & DISTANTE, V. (1986). New trends in the surgical treatment of primary breast cancer in the Florence district: 1979–1985. *Tumori*, **72**, 581.

CONSENSUS CONFERENCES (1985). Adjuvant chemotherapy for breast cancer. *JAMA*, **254**, 3462.

EDITORIAL (1984). Review of mortality results in randomised trials in early breast cancer. *Lancet*, **ii**, 1205.

FISHER, B., BAUER, M., MARGOLESE, R. & 16 others (1985). Five-year results of a randomized clinical trial comparing total mastectomy and segmental mastectomy with or without radiation in the treatment of breast cancer. *N. Engl. J. Med.*, **312**, 665.

GAZET, J.-C., RAINSBURY, R.M., FORD, H.T., POWLES, T.J. & COOMBES, R.C. (1985). Survey of treatment of primary breast cancer in Great Britain. *Br. Med. J.*, **290**, 1793.

GIVIO (Interdisciplinary Group of Cancer Care Evaluation) (1986*a*). Diagnosis and first-line treatment of breast cancer in Italian general hospitals. *Tumori*, **72**, 273.

GIVIO (Interdisciplinary Group for Cancer Care Evaluation) (1986*b*). What doctors tell patients with breast cancer about diagnosis and treatment: Findings from a study in general hospitals. *Br. J. Cancer*, **54**, 319.

GREENBERG, E.R. & STEVENS, M. (1986). Recent trends in breast surgery in the United States and United Kingdom. *Br. Med. J.*, **292**, 1487.

HARRIS, J.R., BEADLE, G.F. & HELLMAN, S. (1984). Clinical studies on the use of radiation therapy as primary treatment of early breast cancer. *Cancer*, **53**, 705.

HARRIS, J.R., HELLMAN, S. & KINNE, D.W. (1985). Limited surgery and radiotherapy for early breast cancer. *N. Engl. J. Med.*, **313**, 1365.

HIMEL, H.N., LIBERATI, A., GELBER, R.D. & CHALMERS, T.C. (1986). Adjuvant chemotherapy for breast cancer. A pooled estimate based on published randomized control trials. *JAMA*, **256**, 1148.

LIBERATI, A., PATTERSON, W.B., BIENER, L. & MCNEIL, B.J. (1987). Determinants of physicians' preferences for alternative treatments in women with early breast cancer. *Tumori*, **73**, 601.

MONTAGUE, E.D. (1984). Conservation surgery and radiation therapy in the treatment of operable breast cancer. *Cancer*, **53**, 700.

SCHLESSELMAN, J.J. (1982). Case-control studies: Design, Conduct and Analysis. Oxford University Press: Oxford.

SIMON, R. (1987). Overviews of randomized clinical trials. *Cancer Treat. Rep.*, **71**, 3.

VANA, J., BEDWANI, R., METTLIN, C. & MURPHY, G.P. (1981). Trends in diagnosis and management of breast cancer in the U.S.: From the surveys of the American College of Surgeons. *Cancer*, **48**, 1043.

VERONESI, U., SACCOZZI, R., DEL VECCHIO, M. & 12 others (1981). Comparing radical mastectomy with quadrantectomy, axillary dissection, and radiotherapy in patients with small cancers of the breast. *N. Engl. J. Med.*, **305**, 6.